# Antibiotic resistance of blood cultures in regional and tertiary hospital settings of Tyrol, Austria (2006-2015): Impacts & trends

**DOI:** 10.1371/journal.pone.0223467

**Published:** 2019-10-10

**Authors:** Peter Kreidl, Thomas Kirchner, Manfred Fille, Ingrid Heller, Cornelia Lass-Flörl, Dorothea Orth-Höller

**Affiliations:** 1 Department of Hygiene, Microbiology and Public Health, Medical University of Innsbruck, Innsbruck, Austria; 2 Department of Orthopedics, Hospital St. Vincent, Zams, Austria; University of Illinois College of Medicine, UNITED STATES

## Abstract

Blood stream infections rank among the top seven causes of death of the general population. The aim of our study was to better understand the epidemiology of BSI in order to improve diagnostics and patient outcome. We used retrospective aggregated laboratory data of blood samples received from all public hospitals in Tyrol, Austria between 2006 and 2015. Microorganisms were categorized into obligatory, facultative, unusual pathogens and contaminants. The distribution, the cumulative incidence and antimicrobial susceptibility patterns were compared between the tertiary (TH) and regional peripheral hospitals (PH). Among 256,364 blood samples, 76.1% were from the TH The incidence of obligatory pathogens was 1.7 fold, and up to 3 times higher for facultative, unusual pathogens and contaminants in the TH and increased mainly due to an increase of *E*.*coli*, which was the most common isolated pathogen (n = 2,869), followed by *Staphylococcus aureus* (n = 1,439), *Enterococcus sp*. (n = 953) and *Klebsiella sp*. (n = 816). The distribution of obligatory pathogens differed between the hospital settings: In the TH *Enterococcus sp*. accounted for 40.8% and *E*.*coli* for 70.4%, respectively, whereas in the PH for 25.4% (p<0.0001) and 57.8%, respectively (p<0.0001) Antibiotic resistance of Gram negative bacteria and *Staphylococcus aureus* did not change during the observation period. Carbapenem resistance of *Klebsiella sp*. and vancomycin and linezolid resistance of *Enterococcus faecium* showed a non-significant increase since 2010 in the TH setting. We concluded that the incidence of BSI in TH was higher compared to PH. We observed higher contamination rates in the TH. We could not interpret the data of coagulase negative staphylococci due to lack of clinical data. We strongly recommend enhancement of training on blood culture sampling to decrease the rate of contamination. Due to differences in pathogen distribution and antimicrobial resistance between different hospital settings we recommend separate treatment guidelines for BSI by hospital setting.

## Introduction

Blood stream infections (BSI) are estimated to rank among the top seven causes of death of the general population, with more than 1.2 million reported episodes and 157,000 deaths per year in Europe [1]. Advances in medicine have led to an increased number of immune-compromised hosts, and invasive devices in the outpatient setting are more frequently used, thus influencing the epidemiology of BSI [2–5]. The emergence of multidrug-resistant pathogens and the lack of development of new antimicrobial agents to combat such infections contribute to this emerging public health problem [6, 7]. Geographical divergence of antimicrobial resistance is well known. In Austria multidrug-resistant bacteria under EU/EEA surveillance consistently remain below the European weighted means [4].

For a successful treatment of sepsis patients, a timely and accurate diagnosis is mandatory and blood culture is still the gold standard in laboratory diagnosis [8]. At least two pairs of aerobic and anaerobic bottles with a sufficient amount of blood (10 ml) obtained through venipuncture are recommended. Samples should be forwarded to the laboratory as soon as possible and incubated for at least 5 days [5]. Interpretation of positive blood cultures should be evaluated in context of the clinical picture. Commensals, such as *Corynebacterium sp*. and *Propionibacterium sp*. usually represent contamination, whereas the detection of viridans group streptococci and coagulase-negative staphylococci is more difficult to interpret. Estimates that the latter mentioned bacteria represent true BSI range between 15% to 78% [5]. In contrast, other identified bacteria such as Enterobacteriaceae or non-fermentative Gram-negative bacteria usually represent true BSI.

The aim of this work was to better understand the epidemiology of BSI in order to improve diagnostics and patient outcome. Objectives were to identify differences in the distribution of pathogens and cumulative incidence between the tertiary and peripheral hospitals and to describe epidemiological trends including changes in susceptibility patterns of the most relevant pathogens over a 10 year period.

## Methods

### Design, setting, participants and samples; interventions; definitions and denominators

We used aggregated retrospective data of all identified positive blood cultures between 2006 and 2015 to describe the distribution of pathogens and their susceptibility patterns by hospital setting with a focus on bacterial pathogens.

During the observation period the laboratory software EKM Bactlab [9] was used for administrative purposes in the Laboratory of the Division of Hygiene and Medical Microbiology (HMM) and the software HyBASE^®^ Labor (epiNET AG) for data extraction. Aggregated data were exported to Microsoft Excel 2010 and further analyzed.

Tyrol, one of Austria’s nine provinces (739,139 inhabitants in 2016) comprises one tertiary hospital (TH) with an annual mean of 1,536.9 hospital beds and an annual mean of 497,955.4 admitted patients, and nine peripheral hospitals (PH) with an annual mean of 2,201.7 beds and an annual mean of 895,958.7 admitted patients during the ten year observation period [10]. Microbiological samples from all hospitals were exclusively sent to the HMM which received between 60 and 80 blood culture sets per day for diagnostic purposes. The TH had an all-in contract with the HMM consisting of a pre-arrangement for the remuneration of all laboratory samples. In contrast, the PH had to pay for each individual sample.

Participants were patients from whom blood cultures were obtained during their hospital stay in one of the Tyrolean hospitals during the ten year observation period. Blood cultures were reported having been obtained only when BSI was suspected according to existing guidelines [8].

All blood cultures from the TH and the PH were included in the description of sampling strategies.

For further analysis consecutive positive samples of patients with the same isolated microorganism were excluded.

The study design was a retrospective cohort design, therefore no interventions were planned.

Blood culture diagnostics comprise aerobic and anaerobic samples. We counted each blood culture bottle as single sample (BC) irrespective of the corresponding patients. This denominator was used to assess the sampling strategies in the two different hospital settings.

Patient samples were defined as the first BC (either aerobic or anaerobic bottles) of each individual patient irrespective of the identification of microorganisms.

Three experienced microbiologists categorized the identified microorganisms into five groups:

- **Group 1: Obligatory pathogens (OP):** always classified as causative agent of blood stream infection (BSI) if identified in blood cultures (e.g. *Staphylococcus aureus*, Enterobacteriaceae such as *Escherichia coli*, *Streptococcus pneumoniae*, *Neisseria meningitidis*)
- **Group 2: Facultative pathogens (FP) excluding coagulase negative staphylococci**: contamination or transient bacteremia cannot be excluded (e.g. alpha hemolytic streptococci)
- **Group 3: Facultative pathogens (FP-CNS)—coagulase negative staphylococci**: contamination or transient bacteremia cannot be excluded
- **Group 4: Unusual pathogens**: rarely detected in humans, rarely described in case reports causing bacteremia (e.g. *Actinomyces sp*., *Lactobacillus sp*., *Moraxella sp*., *Ruminococcus gnavus*), clinical information is essential for interpretation of laboratory results
- **Group 5: Contamination:** very high likelihood of not being responsible for infection (e.g. *Bacillus sp*., *Propionibacterium sp*.)

The cumulative incidence was defined as the cumulative number of positive PS per hospital category divided by number of hospital beds per hospital category.

### Laboratory investigation

The HMM is certified by ISO 9001/2015. Two to three pairs of BC from different sites were recommended to be drawn by venipuncture. More than 90% of samples were forwarded to the HMM the same day. Courier services including transport by ambulances were in place to ensure rapid delivery of samples. The range of delay in samples arriving from the hospitals in the laboratory was considered between 30 minutes and 6 hours, in general. Upon arrival, samples were immediately processed in the BacT/ALERT^®^ 3D microbial detection system (BioMerieux, Marcy-Etoile, France) for all but two peripheral hospitals and BACTEC (Becton Dickinson, Heidelberg, Germany) for the latter. Samples were incubated for five days in exceptional circumstances up to seven days (e.g. HIV patients and suspected endocarditis). As soon as there was an alert of positivity, a Gram-staining and sub-cultivation on agar plates were performed according to the standard techniques [11]. Preliminary positive samples were cultivated on Columbia blood-, chocolate-, MacConkey- and Schaedler anaerobic agar (all Becton Dickinson, Heidelberg, Germany) and incubated for 24 hours at 37°C under aerobic and 48 hours under anaerobic conditions. Identification of pathogens was performed with MALDI^®^ Biotyper system (Bruker Daltonics, Bremen, Germany) using the direct smear method from agar plates since April 2011. Scores above 1.7 were considered valid. In case MALDI-TOF did not deliver appropriate results 16s rRNA gene sequencing was performed. MALDI-TOF MS was exclusively performed from isolates of positive blood cultures. Prior to 2011 biochemical identification using standard microbiological procedures such as API- or VITEK-system (Biomerieux) was conducted.

Antibiotic susceptibility testing was performed according to the European Committee on Antimicrobial Susceptibility Testing (EUCAST) protocol [12] since 2013 and prior according to the CLSI guidelines using disc diffusion testing and the VITEK-system [13, 14].

Positive results of blood cultures of Gram-negative bacteria and yeasts were routinely reported to the sending institution by telephone as soon as a pathogen had been identified by microscopy and the susceptibility results as soon as they were available. All microbiological results were sent to the respective sending institution. The working hours of the laboratory were from 7 a.m. until 7 p.m. including half days on weekends and public holidays.

Data on age, sex, diagnosis, antimicrobial treatment and outcome were not available at HMM. Therefore no infection related outcome could be determined. For retrospective observational studies no ethics committee approval is required by Austrian law.

### Potential threats to internal validity; sample size; statistical methods

All blood culture results were evaluated by a well-trained team of medical microbiologists following the standard operational procedures and 256,364 blood culture samples were investigated.

Potential threats to internal validity were misspelling of names resulting in potential double counting of patients.

Aggregated data were stored and further analysed in excel. The 2-sample z-test was used to compare sample proportions and the Pearson Chi-square test to calculate the trend, using the “EpiTools epidemiological calculators” (Sergeant, ESG, 2018. Epitools epidemiological calculators. Ausvet Pty Ltd. Available at: http://epitools.ausvet.com.au.). A p-value of less than 0.05 was considered statistically significant. Risk ratios including 95% confidence intervals were calculated using MedCalc epidemiological calculator (Ostend, Belgium, 2018. Available at: https://www.medcalc.org/calc/relative_risk.php).

### Ethical statement

All patient samples were stored in an electronic laboratory software in the Laboratory Division of Hygiene and Microbiology. Only anonymous aggregated data were used for analysis. The ethics committee explicitly states that for retrospective data analysis no ethical approval is required.

## Results

**Fig 1:**
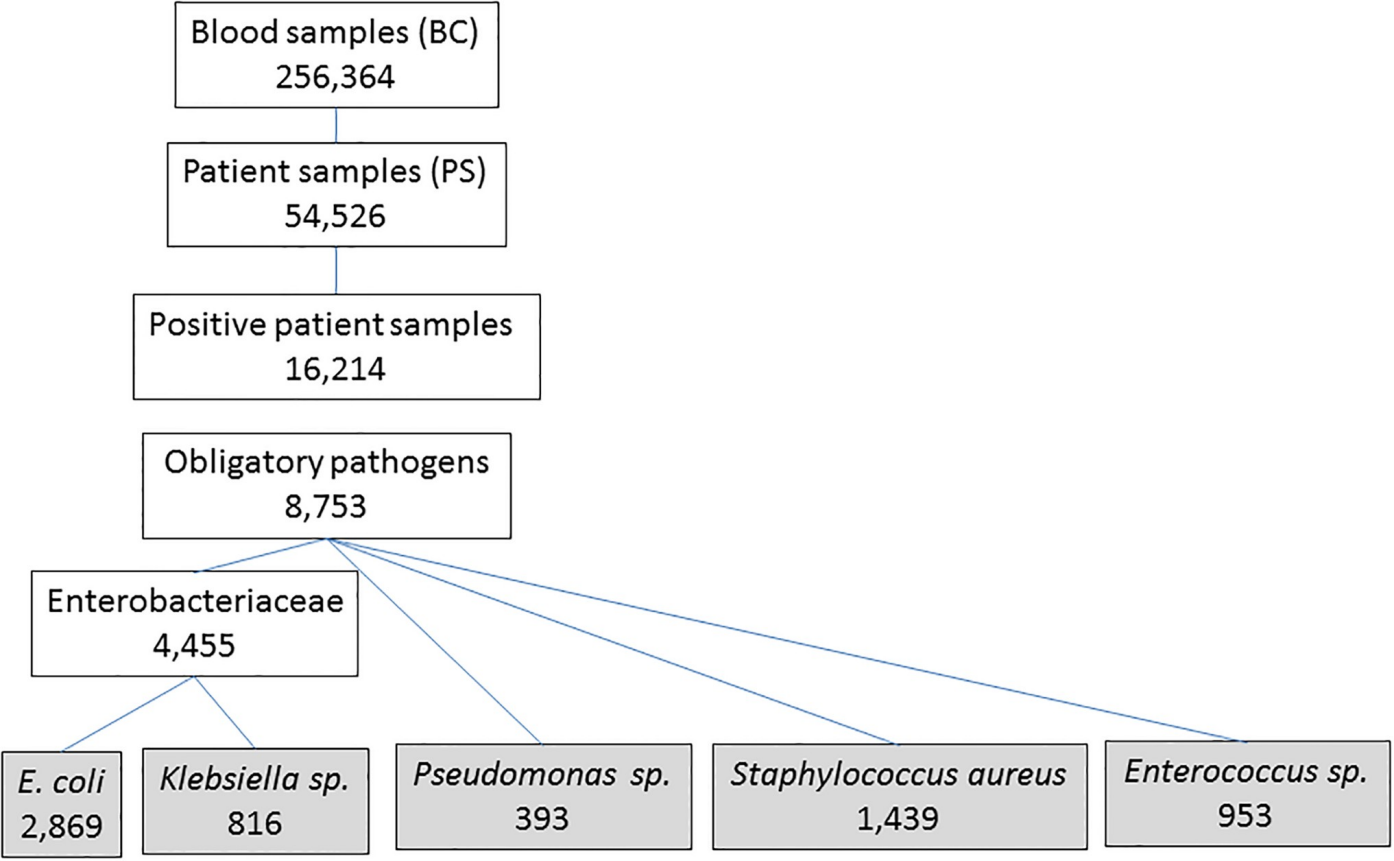
Number of samples and pathogens.

Between 2006 and 2015, we investigated 256,364 BC from Tyrolean hospitals (median 25,285 per year (annual range: 24,160–27,929) (Fig 1).

More than ¾ of samples were sent by the TH. Samples sent from the TH decreased by 19.7% and samples from the PH increased by 44.1% during the observation period.

The mean number of BC per patient was 4.7 (annual range: 4.2–5.4) and higher in the TH (mean 5.6; annual range: 5.1–6.5) compared to PH (mean 3.0; annual range: 2.7–3.3) (p<0.0001). In PH, the median number of BC was increasing from 2.7 to 3.2 per patient, and in contrast, decreasing from 6.1 to 5.4 in the TH.

Among 54,526 patient samples (PS), we identified microorganisms in 29.7% (n = 16,214). This proportion was higher in PH (34.4%, n = 9,292) compared to the TH (27.0%, n = 6,922) (p< 0.0001).

The proportion of PS positive with obligatory pathogens among all PS was also higher in the PH (21.4%) compared to the TH (12.9%; p<0.001).

The cumulative incidence of positive samples per patient was two times higher in the TH (11.2 per 1,000 admitted patients compared to 5.1, in the PH setting and slightly less for obligatory pathogens (5.3 per 1000 admitted patients for TH and 3.2 for PH). Between 2006 and 2015, the annual incidence of obligatory pathogens increased from 4.3 per 1,000 admitted patients to 7.3 in the TH, and from 1.9 to 5.1 in PH, respectively (p<0.001). The risk ratio for a positive result with an obligatory pathogen in a BC was 1.7 times higher in the TH compared to PH. The risk ratios for all other microorganism categories were nearly three times higher in the TH (Table 1).

**Table 1:** Microorganisms, cumulative incidence/1,000 admitted patients and risk ratio (95% confidence interval) by hospital setting.

Microorganism category	N cases TH	% cases TH	Cumulative incidence TH per 1,000 admitted patients	N cases PH	% cases PH	Cumulative incidence PH per 1,000 admitted patients	RR (TH versus PH)	95% CI
Obligatory pathogens	4,443	47.8%	5.3	4,310	62.3%	3.2	1.68	1.61–1.75
Facultative pathogens (without coagulase negative staphylococci)	581	6.3%	1.2	337	4.9%	0.4	2.81	2.46–3.21
Coagulase negative staphylococci	3,808	41.0%	7.6	2,030	29.3%	2.3	3.06	2.90–3.23
Unusual pathogens	120	1.3%	0.2	58	0.8%	0.1	3.37	2.47–4.61
Contamination	340	3.7%	0.7	187	2.7%	0.2	2.96	2.48–3.54
Total	9,292	100%	18.7	6,922	100%	7.7	na	na

na = not applicable; TH = tertiary hospital; PH = peripheral hospitals; CI = confidence interval

### Positive patient samples

More than half of the 16,214 identified microorganisms (54.0%; n = 8,753) were classified as obligatory, 41.7% as facultative (n = 6,756), 1.1% (n = 178) as unusual pathogens and 3.7% (n = 527) as contamination. Eighty six percent of facultative pathogens were due to coagulase negative staphylococci (n = 5,838).

### Obligatory pathogens

Among obligatory pathogens, more than half (58.9%) were Gram-negative bacteria (n = 5,157) of which 99.4% were rods (n = 5,127). Gram-positive bacteria accounted for 33.7% of obligatory pathogens (n = 2,945) of which 97.3% were cocci (n = 2,866). Seven point four percent were fungi (n = 650) of which 99.5% were *Candida sp*. The proportion of fungi was significantly higher in the TH (9.5%; n = 421) compared to PH (5.3%; n = 229) (p<0.0001).

Enterobacteriaceae were the most commonly isolated obligatory pathogens and accounted for 1/3 of obligatory pathogens. *E*.*coli* was the most frequently isolated species followed by *Staphylococcus aureus*, *Enterococcus sp*. and *Klebsiella sp*. in both hospital settings. *E*.*coli*, *Staphylococcus aureus*, *Streptococcus pneumoniae* and beta-hemolytic streptococci were more common in PH, *Enterococcus sp*., *Klebsiella sp*., *Candida sp*., *Pseudomonas sp*. and *Enterobacter sp*. more common in the TH. This distribution of obligatory pathogens remained similar during the observation period. The proportion of *E*. *coli* in the PH increased from 30.9% in 2006 to 41.4% in 2015 (Table 2).

**Table 2:** Number, most commonly isolated pathogens, cumulative incidence/ 1,000 admitted patients by hospital setting.

Pathogen	Total N first patient isolates TH	Cumulative incidence TH per 1,000 admitted patients	% of pathogens TH	Total N first patient isolates PH	Cumulative incidence PH per 1,000 admitted patients	% pathogens PH	P-value difference of % between hospital settings
*E*. *coli*	1,225	1,5	27,6%	1,644	1,2	38,1%	<0.0001
*Staphylococcus aureus*	692	0,8	15,6%	747	0,6	17,3%	0.0319
*Enterococcus sp*.	596	0,7	13,4%	357	0,3	8,3%	<0.0001
*Klebsiella sp*.	469	0,6	10,6%	347	0,3	8,1%	<0.0001
other Gram neg bacteria	379	0,5	8,5%	339	0,2	7,9%	0.770
*Candida sp*.	421	0,5	9,5%	229	0,2	5,3%	<0.0001
*Pseudomonas sp*.	242	0,3	5,4%	151	0,1	3,5%	<0.0001
*Enterobacter sp*.	223	0,3	5,0%	144	0,1	3,3%	<0.0001
*Streptococcus pneumoniae*	78	0,1	1,8%	151	0,1	3,5%	<0.0001
*Streptococcus* non pneumoniae	82	0,1	1,8%	133	0,1	3,1%	<0.0001
Other Gram pos bacteria	36	0,0	0,8%	68	0,1	1,6%	0.735
TOTAL	4,443	5,3	100,0%	4,310	3,2	100%	

TH = tertiary hospital; PH = peripheral hospitals

### Antibiotic susceptibility testing of the most common obligatory pathogens

The risk ratio of pathogens resistant to commonly used antibiotics was higher in the TH for *E*.*coli*, *Klebsiella sp*., *Enterococcus faecium* and *Pseudomonas sp*. Antibiotic resistance of *E*.*coli* and *Klebsiella sp*. to carbapenem was not significantly different between the two hospital settings, and so was cefoxitin resistance (as marker for methicillin resistance) among *Staphylococcus aureus* isolates (Table 3).

**Table 3:** Proportion of resistant strains, cumulative incidence risk ratio (RR) (95% confidence interval).

Pathogen	Antibiotics tested	% resistant TH	% resistant PH	RR (TH versus PH)	95% CI
*E*.*coli*	Aminopenicillin	71.8%	61.4%	1.17	1.11–1.23
	Ciprofloxacin	37.5%	22.7%	1.65	1.47–1.85
	Ceftriaxone	21.3%	13.6%	1.56	1.33–1.84
	Gentamicin	12.1%	6.8%	1.78	1.40–2.24
	Carbapenem	0.3%	0.4%	0.77	0.23–2.62
*Klebsiella sp*.	Ciprofloxacin	26.0%	17.1%	1.53	1.11–2.11
	Ceftriaxone	21.0%	12.8%	1.64	1.13–2.38
	Gentamicin	10.5%	6.3%	3.68	2.22–6.10
	Carbapenem	3.5%	1.6%	2.26	0.74–6.84
*Enterococcus faecium*	Vancomycin	10.1%	2.6%	3.91	1.21–12.60
	Linezolid	5.0%	0.9%	6.12	0.82–46.01
*Enterococcus faecalis*	Vancomycin	2.3%	0.5%	5.07	0.63–40.91
*Staphylococcus aureus*	Cefoxitin*	8.5%	6.8%	1.25	0.87–1.79
*Pseudomonas sp*.	Ciprofloxacin	38.1%	15.1%	2.53	1.66–3.84
	Ceftazidime	26.7%	14.2%	1.88	1.97–2.96
	Gentamicin	26.1%	5.4%	4.80	2.36–9.74
	Carbapenem	38.9%	12.8%	3.03	1.93–4.75
	Piperacillin/tazobactam	19.9%	6.8%	2.93	1.52–5.64

TH = tertiary hospital; PH = peripheral hospitals;

* Cefoxitin as marker for oxacillin/methicillin resistance

**Fig 2:**
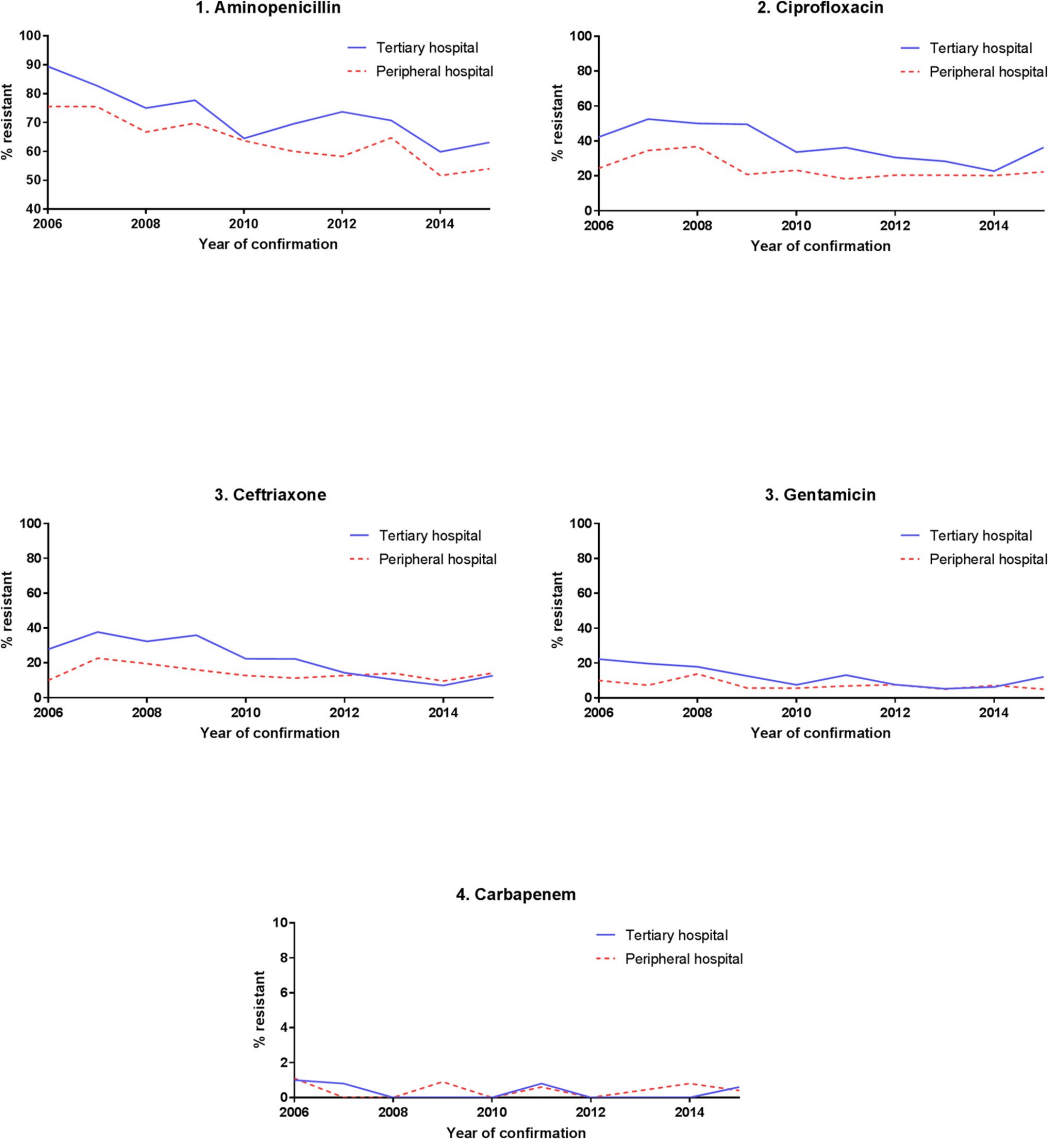
Proportion of antibiotic resistant of E.coli strains over time by hospital setting.

The trend of *E*.*coli* resistance to aminopenicillin and 3^rd^ generation cephalosporin (ceftriaxone) was decreasing during the observation period and undulating for the other tested antibiotics. Between 2014 and 2015, an increase was observed for all tested antibiotic classes (Fig 2).

**Fig 3:**
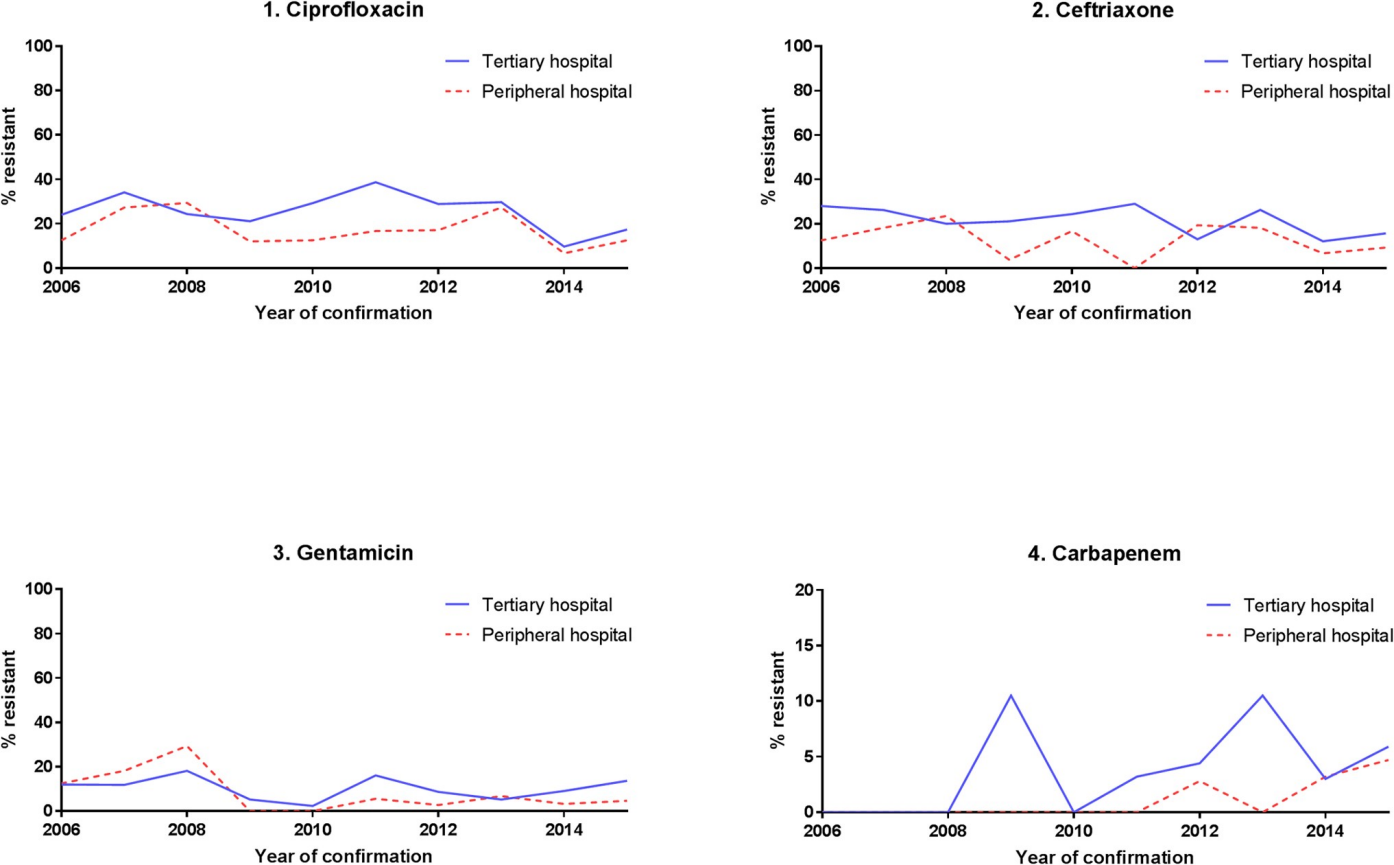
Proportion of antibiotic resistant *Klebsiella sp*. strains over time by hospital setting.

Resistance of *Klebsiella* sp. to tested antibiotics did not change during the observation period except for carbapenem resistance, which showed an increasing trend since 2010/11 in both hospital settings (Fig 3).

The proportion of methicillin resistant *Staphylococcus aureus* (MRSA) (tested for cefoxitin) did not increase over time in neither hospital setting.

**Fig 4:**
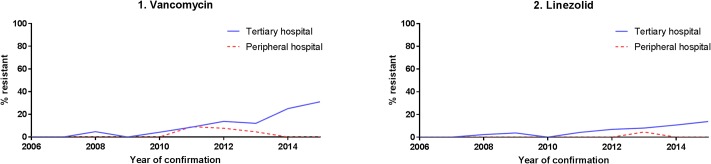
Proportion of antibiotic resistant of *Enterococcus faecium* strains over time by hospital category.

Vancomycin and linezolid resistance of *Enterococcus faecium* increased since 2010 in the TH (Fig 4).

The resistance of *Pseudomonas sp*. to all tested antibiotics remained similar during the observation period.

## Discussion

In our 10 year observational study of blood cultures from all public hospitals in Tyrol, Austria, we found significant differences in the proportion of positive samples, the distribution of pathogens and their resistance patterns between peripheral and the tertiary hospitals. The proportion of positive patient samples and obligatory pathogens was higher in the PH compared to the TH. In addition, the cumulative incidence of positive PS and also that of obligatory pathogens was twice as high in the TH compared to PH, and of facultative-, unusual pathogens and contaminations in the TH even three times higher. This can be attributed to different patient populations between the two hospitals and may also partly be due to a less frequent sampling in the peripheral hospital setting.

Although the TH comprised only 36% of admitted patients compared to the nine PH, ¾ of samples were sent from the TH. In addition, also the mean number of blood culture samples sent per patient was nearly twice as many in the TH compared to the PH. This was partly due to different contracts between the two hospital settings: an all-in contract with the TH and individual payment of each sample from the PH.

In general, health care workers obtaining blood cultures need to be well trained and blood culture collection protocols should be repeatedly emphasized. Feghaly et al. describe lower contamination rates by enhancing training and standardizing protocols [15]. Kirn et. al. conclude that increasing the number of samples without enhancing the quality of blood culture collection will result in higher contamination rates, unnecessary antibiotic treatment, longer hospital stays and higher costs [5]. Current guidelines suggest that six bottles (3 pairs of each 10 mL) of blood cultures taken by venipuncture within 24 hours are needed to reach a sensitivity of 95–99% in detecting bacteremia [8] only from patients presenting with clinical findings compatible with blood stream infections due to low circulating viable pathogens [5, 16]. Lack of adequate training on blood sampling techniques may also have contributed to the high contamination rates, especially in the tertiary hospital setting.

Rodriguez-Bano et. al. found that 58% of BSI were hospital-acquired infections, 24% were healthcare associated and 18% were community acquired in two similar hospital settings [17]. As our data did not allow a distinction between the above mentioned categories we can only assume that the distribution between community-, healthcare-associated and hospital-acquired infection might be similar. Even though our data did not allow stratification between these categories, we found a significant difference in the number of BSI episodes between TH and PH hospital settings. More severe conditions of patients, a higher proportion of immunocompromised patients and probably a more frequent use of broad spectrum antibiotics were likely to have increased the risk of infection and antimicrobial resistance in the TH.

The detailed analysis of obligatory pathogens revealed *E*. *coli* as most frequently isolated pathogen, accounting for approximately one third of obligatory pathogens, followed by *Staphylococcus aureus* which was isolated approximately half as frequently compared to *E*.*coli*.

The likelihood of detection of certain pathogens varied significantly between the two hospital settings: while *E*.*coli*, *Staphylococcus aureus*, *Streptococcus pneumoniae* and *beta hemolytic streptococci* were observed more frequently in the PH, *Enterococcus sp*., *Klebsiella sp*. and *Enterobacter sp*. were more frequently isolated from patients admitted to the TH. A similar distribution of obligatory pathogens—*E*. *coli* as most common isolated pathogen followed by *Staphylococcus aureus—* was described by several authors [18–21]. Comparisons with international data remain limited due to different inclusion criteria of pathogens.

*E*.*coli* showed an increasing trend mainly in the peripheral hospitals while *Staphylococcus aureus* remained stable in both hospital settings. An increasing trend of *E*. *coli* BSI was also described in France [22] and Finland [23] mainly among 3^rd^ generation cephalosporin resistant strains. Van der Mee-Marquet et. al. concluded, that the increase of community acquired BSI due to *E*.*coli* can be greatly attributed to an increase in persons > 74 years of age [22]. According to the authors, immune senescence, changes in mucosal and skin barriers, degenerative changes and comorbid conditions are contributing factors in elderly patients [22]. Unfortunately, age was not available for our data analysis.

Antibiotic resistance to all tested antibiotics was consistently higher in the TH, although due to small numbers not all results were statistically significant.

The decrease in antibiotic susceptibility of 3^rd^ generation cephalosporins was probably partly caused by the switch from CLSI (Clinical and Laboratory Standards Institute) guidelines to EUCAST in February 2012 [24]. Prior to the recommendation of EUCAST extended spectrum beta-lactamase producing enterobacteriaceae were always classified as 3^rd^ generation cephalosporin resistant. Since then, resistance to the above mentioned antibiotics has been reported according to the zone of inhibition [25].

We conclude that guidance for empirical antibiotic treatment of BSI should be tailored according to the hospital setting due to the different susceptibility patterns in the two hospital settings.

The emergence of vancomycin and linezolid resistant enterococci and carbapenem resistance in Gram-negative bacteria needs close monitoring and rigorous antimicrobial stewardship.

One main limitation of our study was that we were lacking detailed patient information. Therefore it was neither possible to distinguish nosocomial from community acquired infections nor to interpret the relevance of isolated coagulase negative staphylococci. In our available aggregated dataset, we classified all coagulase negative staphylococci as facultative pathogens. We could not apply any criteria with the available data, which would have allowed us to distinguish between infection and contamination and thus we decided not to further interpret CNS positivity. According to literature [5, 26] we have to assume that at least 15% of the positive samples with confirmed CNS were real BSI. Therefore, our classification of pathogens probably resulted in an underestimation of the true burden of BSI. In addition, we were not able to assess if there was any difference of BSI of CNS positive blood samples by hospital setting.

Nevertheless, the close collaboration and discussions with the tertiary hospital staff helped in individually interpreting the clinical importance of blood cultures found positive for CNS at the time of diagnosis.

Smith et. al. reported previous linezolid exposure and duration as main determinants for development of linezolid resistance in enterococci [27]. In the TH the linezolid usage was high ranging from 8,000 to 10,500 grams per. No further molecular analysis to establish the mechanism of linezolid resistance was conducted in our isolates.

Furthermore, we were not able to calculate the population based cumulative incidence as the catchment population of the TH consisted of patients both, from one district but also referrals from the PH.

Two peripheral hospitals, which accounted for less than 5% of admitted patients in the PH only sent positive blood cultures for species diagnosis and antibiotic susceptibility testing which resulted in an overestimation of the proportion of positive samples from PH. Nevertheless, this is unlikely to have influenced the calculated cumulative incidence, which was similar to published results [28].

Despite the important limitations of our study which hamper our analysis, we are confident that our observations are important also for other settings. Since 2017, we have implemented a new computerized laboratory surveillance which will allow case based analysis as more detailed patient information is available for analysis. The new system also allows cluster detection and thus timely response to potential outbreaks.

General practitioners and rehabilitation centers were not included in our study which may have biased our results.

The fact that we could not control for BSI with multiple pathogens resulted in an overestimation of proportion of positive blood cultures per patient compared to the literature (16.1% versus 10.9%) [29]. An analysis of BSI during 2017 revealed that in 7.1% of positive blood cultures more than one pathogen was identified.

In summary we conclude, that the cumulative Incidence of BSI in TH is higher compared to PH. In addition, we also observed higher contamination rates in the TH. Therefore, we strongly recommend enhancement of training on blood culture sampling to decrease the rate of contamination especially in settings where blood culture sampling is frequently conducted. Due to the significant differences in pathogen distribution and resistance patterns between the two hospital settings, we suggest an adaptation of the current recommendation of empiric treatment for BSI by hospital setting. Furthermore, the emergence of multiresistant pathogens, such as vancomycin and linezolid resistant *Enterococci* and carbapenem resistant *Klebsiella* needs special attention and rigorous antimicrobial stewardship.

## Supporting information

S1 Table Microorganisms, cumulative incidence/1,000 admitted patients and risk ratio (95% confidence interval) by hospital setting. (PDF)

S2 Table Number, most commonly isolated pathogens, cumulative incidence/ 1,000 admitted patients by hospital setting. (PDF)

S3 Table Proportion of resistant strains, cumulative incidence risk ratio (RR) (95% confidence interval). (PDF)

S1 Appendix Aggregated data. (XLSX)
