# Supplementary material for: Antibiotic resistance of blood cultures in regional and tertiary hospital settings of Tyrol, Austria (2006-2015): Impacts & trends
Source: PLoS One. 2019 Oct 10;14(10):e0223467. doi: 10.1371/journal.pone.0223467 (PMC6786751; doi:10.1371/journal.pone.0223467)
Supplement: S1 Table — (PDF) [file pone.0223467.s001.pdf]

Table 1: Microorganisms, cumulative incidence/1,000 admitted patients and risk ratio (95% confidence interval) by hospital setting

Microorganism category	N cases TH	% cases TH	Cumulative incidence TH per 1,000 admitted patients	N cases PH	% cases PH	Cumulative incidence PH per 1,000 admitted patients	RR (TH versus PH)	95% CI
Obligatory pathogens	4,443	47.8%	5.3	4,310	62.3%	3.2	1.68	1.61-1.75
Facultative pathogens (without coagulase negative staphylococci)	581	6.3%	1.2	337	4.9%	0.4	2.81	2.46-3.21
Coagulase negative staphylococci	3,808	41.0%	7.6	2,030	29.3%	2.3	3.06	2.90-3.23
Unusual pathogens	120	1.3%	0.2	58	0.8%	0.1	3.37	2.47-4.61
Contamination	340	3.7%	0.7	187	2.7%	0.2	2.96	2.48-3.54
Total	9,292	100%	18.7	6,922	100%	7.7	na	na

Legend: na=not applicable; TH = tertiary hospital; PH= peripheral hospitals; CI= confidence interval
