# Supplementary material for: Antibiotic resistance of blood cultures in regional and tertiary hospital settings of Tyrol, Austria (2006-2015): Impacts & trends
Source: PLoS One. 2019 Oct 10;14(10):e0223467. doi: 10.1371/journal.pone.0223467 (PMC6786751; doi:10.1371/journal.pone.0223467)
Supplement: S2 Table — (PDF) [file pone.0223467.s002.pdf]

Table 2: Number, most commonly isolated pathogens, cumulative incidence/ 1,000 admitted patients by hospital setting

Pathogen	Total N first patient isolates TH	Cumulative incidence TH per 1,000 admitted patients	% of pathogens TH	Total N first patient isolates PH	Cumulative incidence PH per 1,000 admitted patients	% pathogens PH	P-value difference of % between hospital settings
E. coli	1,225	1,5	27,6%	1,644	1,2	38,1%	<0.0001
Staphylococcus aureus	692	0,8	15,6%	747	0,6	17,3%	0.0319
Enterococcus sp.	596	0,7	13,4%	357	0,3	8,3%	<0.0001
Klebsiella sp.	469	0,6	10,6%	347	0,3	8,1%	<0.0001
other Gram neg bacteria	379	0,5	8,5%	339	0,2	7,9%	0.770
Candida sp.	421	0,5	9,5%	229	0,2	5,3%	<0.0001
Pseudomonas sp.	242	0,3	5,4%	151	0,1	3,5%	<0.0001
Enterobacter sp.	223	0,3	5,0%	144	0,1	3,3%	<0.0001
Streptococcus pneumoniae	78	0,1	1,8%	151	0,1	3,5%	<0.0001
Streptococcus non pneumoniae	82	0,1	1,8%	133	0,1	3,1%	<0.0001
Other Gram pos bacteria	36	0,0	0,8%	68	0,1	1,6%	0.735
TOTAL	4,443	5,3	100,0%	4,310	3,2	100%	

Legend: TH = tertiary hospital; PH= peripheral hospitals
