# Supplementary material for: Antibiotic resistance of blood cultures in regional and tertiary hospital settings of Tyrol, Austria (2006-2015): Impacts & trends
Source: PLoS One. 2019 Oct 10;14(10):e0223467. doi: 10.1371/journal.pone.0223467 (PMC6786751; doi:10.1371/journal.pone.0223467)
Supplement: S3 Table — (PDF) [file pone.0223467.s003.pdf]

Table 3: Proportion of resistant strains, cumulative incidence risk ratio (RR) (95% confidence interval)

Pathogen	Antibiotics tested	% resistant TH	% resistant PH	RR (TH versus PH)	95% CI
E.coli	Aminopenicillin	71.8%	61.4%	1.17	1.11-1.23
	Ciprofloxacin	37.5%	22.7%	1.65	1.47-1.85
	Ceftriaxone	21.3%	13.6%	1.56	1.33-1.84
	Gentamicin	12.1%	6.8%	1.78	1.40-2.24
	Carbapenem	0.3%	0.4%	0.77	0.23-2.62
Klebsiella sp.	Ciprofloxacin	26.0%	17.1%	1.53	1.11-2.11
	Ceftriaxone	21.0%	12.8%	1.64	1.13-2.38
	Gentamicin	10.5%	6.3%	3.68	2.22-6.10
	Carbapenem	3.5%	1.6%	2.26	0.74-6.84
Enterococcus faecium	Vancomycin	10.1%	2.6%	3.91	1.21-12.60
	Linezolid	5.0%	0.9%	6.12	0.82-46.01
Enterococcus faecalis	Vancomycin	2.3%	0.5%	5.07	0.63-40.91
Staphylococcus aureus	Cefoxitin*	8.5%	6.8%	1.25	0.87-1.79
Pseudomonas sp.	Ciprofloxacin	38.1%	15.1%	2.53	1.66-3.84
	Ceftazidime	26.7%	14.2%	1.88	1.97-2.96
	Gentamicin	26.1%	5.4%	4.80	2.36-9.74
	Carbapenem	38.9%	12.8%	3.03	1.93-4.75
	Piperacillin/tazobactam	19.9%	6.8%	2.93	1.52-5.64

*Legend: TH = tertiary hospital; PH= peripheral hospitals; * Cefoxitin as marker for oxacillin/methicillin resistance*
